# Arbuscular mycorrhizal fungi enhanced resistance to low-temperature weak-light stress in snapdragon (*Antirrhinum majus* L.) through physiological and transcriptomic responses

**DOI:** 10.3389/fpls.2024.1330032

**Published:** 2024-04-12

**Authors:** Wei Li, Haiying Wu, Junkai Hua, Chengshang Zhu, Shaoxia Guo

**Affiliations:** ^1^ Country College of Landscape Architecture and Forestry, Qingdao Agricultural University, Qingdao, Shandong, China; ^2^ Institute of Mycorrhizal Biotechnology, Qingdao Agricultural University, Qingdao, Shandong, China

**Keywords:** *Antirrhinum majus*, arbuscular mycorrhizal fungi, low temperature, weak light, physiology, transcriptome

## Abstract

**Introduction:**

Low temperature (LT) and weak light (WL) seriously affects the yield and quality of snapdragon in winter greenhouse. Arbuscular mycorrhizal fungi (AMF) exert positive role in regulating growth and enhancing abiotic stress tolerance in plants. Nevertheless, the molecular mechanisms by AMF improve the LT combined with WL (LTWL) tolerance in snapdragon remain mostly unknown.

**Methods:**

We compared the differences in root configuration, osmoregulatory substances, enzymatic and non-enzymatic antioxidant enzyme defense systems and transcriptome between AMF-inoculated and control groups under LT, WL, low light, and LTWL conditions.

**Results:**

Our analysis showed that inoculation with AMF effectively alleviated the inhibition caused by LTWL stress on snapdragon root development, and significantly enhanced the contents of soluble sugars, soluble proteins, proline, thereby maintaining the osmotic adjustment of snapdragon. In addition, AMF alleviated reactive oxygen species damage by elevating the contents of AsA, and GSH, and the activities of superoxide dismutase (SOD), peroxidase (POD), catalase (CAT), ascorbate peroxidase (APX), dehydroascorbate reductase (DHAR), monodehydroascorbate reductase (MDHAR), and glutathione reductase (GR). RNA-seq analysis revealed that AMF regulated the expression of genes related to photosynthesis (photosystem I related proteins, photosystem II related proteins, chlorophyll a/b binding protein), active oxygen metabolism (POD, Fe-SOD, and iron/ascorbate family oxidoreductase), plant hormone synthesis (*ARF5* and *ARF16*) and stress-related transcription factors gene (*bHLH112, WRKY72, MYB86, WRKY53, WRKY6*, and *WRKY26*) under LTWL stress.

**Discussion:**

We concluded that mycorrhizal snapdragon promotes root development and LTWL tolerance by accumulation of osmoregulatory substances, activation of enzymatic and non-enzymatic antioxidant defense systems, and induction expression of transcription factor genes and auxin synthesis related genes. This study provides a theoretical basis for AMF in promoting the production of greenhouse plants in winter.

## 1 Introduction

In recent years, global climate change has led to the frequent exposure of plants to a variety of abiotic stresses (Jambunathan et al., 2009; Sham et al., 2014). Snapdragon (*Antirrhinum majus* L.), a member of the family Basalaceae native to the Mediterranean region, is a perennial flowering plant often regarded as an annual by horticulturists (Carter and Grieve, 2008). Because of its bright colors and beautiful flower patterns, it is widely used in flower beds. The optimal growth temperature of snapdragon is 15–16°C. When temperatures drop below 5°C, snapdragon can experience cold damage, resulting in flowering failure or even death (Sharma et al., 2018). Weak light (WL) stress inhibits anthocyanin synthesis in snapdragon and has an impact on flowering quality (Ichimura et al., 2021). Therefore, it is urgent to study the effects of low temperature and weak light stress (LTWL) on physiological function of snapdragon and the breeding of LTWL resistant varieties.

Most previous studies involving low temperature (LT) or WL have been in the form of individual stress, while LTWL stress has been less studied. Studies have shown that LT stress inhibits photosynthetic efficiency and PSII activity of plants (Hikosaka et al., 2006; Höglind et al., 2011), reduces membrane fluidity, leading to low water availability (Ma et al., 2018), resulting in increased membrane permeability, accompanied by electrolyte leakage and significant changes in structure and lipid composition, these changes lead to high concentrations of reactive oxygen species (ROS), including superoxide anion, hydrogen peroxide, and hydroxyl radical production (Li et al., 2015), which in turn destroys cellular lipids, proteins, DNA, and other macromolecules (Chen et al., 2014), as well as enzyme inhibition, and programmed cell death, which can eventually lead to plant death (Mishra et al., 2011; Pessarakli, 2016).

Furthermore, the change of light intensity directly affects the growth characteristics and plant metabolism. WL leads to the reduction of photosynthetic pigments, hinder electron transport in the photosystem, reduce the activity of carbon assimilation enzymes (Hu et al., 2017), and result in significant reductions of proline, the increase of ROS and malondialdehyde (MDA), lead to changes in a series of antioxidant enzyme activities and osmotic regulators (Meng et al., 2017; McLay et al., 2018), root activity decreased, and the development of the palisade and spongy tissues of leaves slowed down (Ou et al., 2015; Yang et al., 2016).

However, temperature and light are usually two related environmental factors (Legris et al., 2017), and it is clear that simultaneous exposure to multiple stressors can have more serious consequences than exposure to single factor stressors, because the effects of combined stressors can have synergistic effect on plants, acting through complex mechanisms, and cannot be predicted by adding the effects of two single-factor stressors (Teige et al., 2004; Sewelam et al., 2014; Suzuki et al., 2014). In fact, at the molecular level, interactions among signaling pathways have been observed in response to LTWL (Janda et al., 2014; Yan et al., 2013). It was found that LT and WL stress caused damage to cell membrane, increased osmotic substances, weakened photosynthesis, damaged leaf tissue (Tang et al., 2021), membrane peroxidation, reduced biomass, decreased photosynthetic capacity (Li et al., 2015), antioxidant accumulation (Genzel et al., 2021), and down-regulated expression of key genes.

The decrease of antioxidant enzyme activity can also cause gray mold, affecting plant growth and fruit quality (Fan et al., 2017). Due to the limited sunshine time in winter in cities in northern China, the temperature in plastic greenhouses is often quite low, so LTWL events often occur in the restricted production of plastic greenhouses and facility agriculture (Yang et al., 2016; Xu et al., 2020), the traditional solution is to install passive climate control systems in greenhouses when crops experience LTWL stress, but installation costs are high (Piñero et al., 2022). It has been widely reported that AMF positively affects plant growth by promoting soil aggregation, soil nutrient cycling, reducing nutrient loss, nutrient uptake, and biotic and abiotic stress tolerance (Smith and Smith, 2011; Bender et al., 2016). Studies found that AMF inoculation improved the growth condition of *Zea mays* L. (Zhu et al., 2010), *Oryza sativa* L. (Liu et al., 2013), and *Cucumis sativus* L. (Chen et al., 2013) under LW stress. In addition, under WL stress, AMF inoculation can maintain the high activity of antioxidant enzymes such as superoxide dismutase (SOD), catalase (CAT) and ascorbate peroxidase (APX), as well as the high content of proline, promoting the growth of tall fescue (Zhang et al., 2019b). AMF inoculation can alleviate the adverse effects of shade on plants to a certain extent (Shukla et al., 2018).

It is well known that the establishment of a symbiotic relationship between AMF and plant roots leads to changes in the transcriptome of the host plant (Bucher et al., 2014), which brings multiple benefits to the plant in terms of growth, development, and stress tolerance. However, to the best of our knowledge, few studies have investigated how AMF host plants change under LTWL conditions at the transcriptional level, which further limits the application of AMF for improving plant stress tolerance. Therefore, in this study, we hypothesized that AMF may enhance snapdragon’s tolerance to LTWL by modulating the antioxidant defense system and other physiological processes, as well as changes in the transcriptome level. Based on the clear physiological parameters of mycorrhizal snapdragon, this study identified the key genes of mycorrhizal snapdragon in response to LT, WL and LTWL stress through transcriptome sequencing, and initially understood the oxidative defense mechanism of snapdragon in response to LTWL stress. This study will be conducive to the cultivation of snapdragon.

## 2 Materials and methods

### 2.1 Experimental material

*Antirrhinum majus* ‘Red and White’ seeds were purchased from Taki Seedlings Co., Ltd for use in the experiments. The seeds were sterilized with 3% sodium hypochlorite for 10 min and washed three times using sterile water. The seeds were germinated on wet filter paper in plates at 25°C for 3 days.

The AMF species *Funneliformis mosseae* and *Glomus versiforme* were provided by the Mycorrhizal Biotechnology Research Institute of Qingdao Agricultural University. The two AMF species were propagated on clover (*Trifolium repens* L.) as a host plant in 5L pots containing sterile sandy loam soil, under greenhouse conditions. Hoagland solution (phosphate concentration 25%) prepared with deionized water was added to the pots weekly. After 4 months, plant shoots were cut off, and pot materials containing soil, fungal spores, mycelium, and mycorrhizal root segments of host plants were thoroughly mixed and used as fungal inoculum. Mycorrhizal colonization of root fragments in inoculum was 50% for *Funneliformis mosseae* and 50% for *Glomus versiforme*.

The soil used in the experiment was a mixture of garden soil and peat (1:1, v: v). The garden soil was collected from the campus of Qingdao Agricultural University (Shandong Province China). The soil was sieved by passing it through a 2 mm mesh, and autoclaved (120°C, 2 h) for 2 h. The pH of the soil was 6.6 (1:2.5 water), and the soil contained with organic matter 8.6 g·kg^-1^, total nitrogen 0.86 g·kg^-1^, total phosphorus 0.50 g·kg^-1^, total potassium 9.7 g·kg^-1^, hydrolyzable nitrogen 44 mg·kg^-1^, available phosphorus 10.1 mg·kg^-1^, available potassium 65.4 mg·kg^-1^.

### 2.2 Experimental design

The experiment was conducted in the artificial climate chamber of Qingdao Agricultural University from January to March 2020. The experiment was divided into inoculation and non-inoculation groups. After seeds germination three days, one germinated snapdragon seeds were sowed into a plastic pot (height × upper diameter × lower diameter = 24 cm × 24 cm × 18 cm) containing 2.5 kg of autoclaved soil. 50 g of AM fungal inoculum (5000 inoculum potential units) was added to each pot for the inoculation treatment, and 50 g of sterilized inoculum was added to the non-inoculation control. The inoculum was applied approximately 2 cm below the seeds. The pots were placed randomly in a block design. Then the plants were grown under control conditions in an artificial climate chamber. The day/night temperature was 25/15°C, with the relative humidity of 70% and the photoperiod of 12/12 h day/night under a photon flux density of about 500 μmol·m^-2^·s^-1^. The pots were regularly rotated within and among the cultivation shelves to minimize the effects of the light intensity differences. Plants were watered every second day. Hoagland solution (phosphate concentration 25%) prepared with deionized water was added to the pots weekly.

After 11 weeks of cultivation, the snapdragon seedlings from each groups were randomly divided among four treatments, resulting in 8 total treatment groups: 1) LT, non-inoculated plant at 14/4°C, 500 μmol·m^-2^·s^-1^ light intensity; 2) LTI, inoculated plant at 14/4°C, 500 μmol·m^-2^·s^-1^ light intensity; 3) WL, non-inoculated plant at 25/15°C, light intensity of 100 μmol·m^-2^·s^-1^; 4) WLI, inoculated plant at 25/15°C, light intensity of 100 μmol·m^-2^·s^-1^; 5) LTWL, non-inoculated plant at 14/4°C, light intensity 100 μmol·m^-2^·s^-1^; 6) LTWLI, inoculated plant at 14/4°C, light intensity 100 μmol·m^-2^·s^-1^; 7) NTNL, non-inoculated plant at 25/15°C, light intensity 500 μmol·m^-2^·s^-1^; 8) NTNLI, inoculated plant at 25/15°C, light intensity 500 μmol·m^-2^·s^-1^. And the photoperiod of the above treatments were 12h/12h light-dark cycle. After one week of treatment, nine plants with similar growth activities were selected from each treatment and grouped into three biological replicates (three plants each). All samples were immediately placed in liquid nitrogen and stored at -80°C for future use.

### 2.3 Root configuration and physiological indexes

The roots of snapdragon were collected and any attached soil was washed away with slow running water, any roots that drew in water were separated. The root system was scanned with a scanner. Root length, total root surface area, total root volume, mean root diameter, number of root tips, and number of root forks were determined using the root analysis system (Win RHIZO-Pro 2008b, Canada).

After snapdragon treated for 7 d, 0.5 g of fresh leaves from different plants were weighed into a mortar, and 10 mL phosphoric acid buffer (0.05 mol/L, pH 7.8) was added; the mixture was then ground in an ice bath and homogenized at -4°C. After centrifugation at 4,000 rpm for 20 min, the supernatant was used to determine the enzyme activities. The relative conductivity was determined as described previously (Dionisio-Sese and Tobita, 1998), soluble sugar content was determined using the phenol method (Buysse and Merckx, 1993), and soluble protein content was determined using the Coomassie blue G-250 method (Bradford, 1976). Proline was determined using acid ninhydrin colorimetry (Bates et al., 1973). SOD was determined by the NBT method (Constantine and Stanley, 1977), POD was determined using the guaiacol method (Chance and Maehly, 1955). CAT activity was determined according to Cakmak and Marschner (1992). The activity of ascorbate peroxidase (APX) was determined according to Amako et al. (1994) formula, and the activity of glutathione reductase (GR) was determined according to Edwards et al. (1990) method. DHAR activity was determined according to Nakano and Asada (1981). The activity of MDHAR was determined according to Krivosheeva et al. (1996), the contents of AsA and oxidized ascorbic acid (DHA) were determined according to Costa et al. (2002), and the contents of GSH and GSSG were determined according to Griffith (1980), and the lipid peroxidation was estimated from the MDA content, which was determined using the TBA method (Velikova et al., 2000).

### 2.4 Total RNA extraction, library construction, and sequencing

Total RNA from snapdragon leaves was extracted using the TRIzol (Invitrogen, CA, USA) method. The total RNA quantity and purity were analysis of Bioanalyzer 2100 and RNA 1000 Nano LabChip Kit (Agilent, CA, USA) with RIN number >7.0. Poly(A) RNA was purified from total RNA (5 μg) using poly-T oligo-attached magnetic beads using two rounds of purification. Following purification, the mRNA was fragmented into small pieces using divalent cations under elevated temperature. Then the cleaved RNA fragments were reverse-transcribed to create the final cDNA library in accordance with the protocol for the RNA-Seq sample preparation kit (Illumina, San Diego, USA), the average insert size for the paired-endlibraries was 300 bp ± 50 bp. Finally, we used the Illumina Novaseq™ 6000 (LC Biotechnology CO., Ltd. Hangzhou, China) to conduct double-end sequencing in the PE150 mode as per standard procedure. The sequencing data has been submitted to the NCBI Sequence Read Archive database (Accession number: PRJNA643012, PRJNA643405, PRJNA643675, PRJNA644043 and PRJNA644272).

### 2.5 Alignment with reference genome sequence

The Cutadapt 1.9 (https://cutadapt.readthedocs.io/en/stable/) software was used to remove the reads that contained adaptor contamination, low quality bases, and undetermined bases (Martin, 2011). We used HISAT2.2.0.4 software (https://daehwankimlab.github.io/hisat2/) (Kim et al., 2015) to map reads to the snapdragon reference genome (http://bioinfo.sibs.ac.cn/Am) (Li et al., 2019).

### 2.6 Functional annotation and enrichment analysis of differentially expressed genes

Mapping of each sample reading was conducted using the StringTie 1.3.4 software (http://ccb.jhu.edu/software/stringtie/) at the beginning of transcriptome assembly. The gffcompare 0.9.8 software (http://ccb.jhu.edu/software/stringtie/gffcompare.shtml) was used to compare the transcript with the reference comment to get the final assembly comment result. Based on the comparison results, StringTie was used to determine the mRNA expression levels by calculating FPKM (FPKM = [total_exon_fragments/mapped_reads(millions) × exon_length(kB)]). The differentially expressed genes (DEGs) were selected with |log2FoldChange| > 1 and *p* < 0.05 by R package edgeR(https://bioconductor.org/packages/release/bioc/html/edgeR.html) (Robinson et al., 2010 or DESeq2(http://www.bioconductor.org/packages/release/bioc/html/DESeq2.html) (Pertea et al., 2015), and then analysis GO enrichment and KEGG enrichment to the DEGs. The DEGs were matched using BLAST analysis against a variety of protein databases, including NCBI non-redundant nucleotide sequences (Nt), NCBI non-redundant protein sequences (NR), protein family (Pfam), manual annotation, reviewed protein sequence (Swiss-Prot), and the Clusters of Orthologous Groups of proteins (KOG/COG). Principal Component Analysis (PCA) plots were ggplot2 packages in R software to study the similarity or difference of sample community composition (Villanueva and Chen, 2019).

### 2.7 Real-time reverse transcription PCR

To evaluate gene expression levels, we carried out quantitative reverse transcription-polymerase chain reaction (qRT-PCR). RNA samples (containing approximately 1 µg total RNA) were treated with gDNA Eraser to eliminate any contaminant gDNA. qRT-PCR was performed using the ABI StepOnePlus PCR System (ABI, USA) with the SYBR^®^ Premix Ex Taq TM^II^ kit (TaKaRa, Dalian, China). The melting curves of candidate genes and reference genes were analyzed, and A single amplification product of the expected size for each gene was verified by electrophoresis on a 1.5% (m/V) agarose gel (100 V) electrophoresis for 25 min. The cDNA templates of all samples were taken in equal amounts, and the amplification efficiency were derived from the relative standard curve for the transcripts that were generated with a serial dilution of cDNA (1, 10^-1^, 10^-2^, 10^-3^, 10^-4^, 10^-5^, 10^-6^). The standard curve was drawn according to the obtained Ct values, and the slope K and linear correlation coefficient (R^2^) were obtained. The amplification efficiency E was calculated according to the formula E= (10^(-1/K)^-1) ×100% (Ginzinger, 2002). The 20-μl qRT-PCR solutions consisted of 10.0 μl 2 × SYBR^®^ Premix Ex Taq™ II, 0.4 μl 50 × ROX reference dye, the forward and reverse primers 0.8 μl (10 µM), 4.0 μl cDNA, and 4.0 μl RNase-Free ddH_2_O. The PCR protocol was as follows: 95°C for 15 min, followed by 40 cycles of 95°C for 10 s, 60°C for 20 s, and 72°C for 30 s, and a final 10 min at 72°C. The *AmUBI* gene (accession: Am03g01530), which was stably expressed according to the RNA sequencing data, was used to normalize expression levels (ManChado-Rojo et al., 2012). The 2^−ΔΔCt^ comparative threshold cycle (Ct) method was used to evaluate gene expression levels (Livak and Schmittgen, 2001), where ΔΔCt = (Ct_1_ -Ct*_AmUBI_*)- (Ct_2_ - Ct*_AmUBI_*). Ct_1_ represents the DEGs Ct values of inoculation with AMF (LTI, WLI and LTWLI), and Ct_2_ represents the DEGs Ct values of non-inoculation groups (LT, WL and LTWL). The primers used in this study for qRT-PCR analysis are listed in **Supplementary Table S1** and three biological replicates were examined for each reaction.

### 2.8 Statistical analysis

The results from this experiment were averaged over 3 biological replicates. Physiological data were analyzed in Microsoft Excel 2016 (Microsoft Company, USA) and statistical analysis was conducted in SPSS (version 18.0, SPSS Institute, USA). Analysis of variance (ANOVA) was used to test for significant differences in means across treatments. When significant differences were found (p < 0.05), multiple comparisons were tested by Duncan’s multiple range test (n = 3) at a 0.05 significance level.

## 3 Results and analysis

### 3.1 Root configuration

Under the condition of NTNL, the total root length, total root surface area, total root projected area, total root volume, average root diameter, root tip number, and root tip bifurcation number were significantly increased in the AMF group (NTNLI) by 34.0%, 31.5%, 38.2%, 29.4%, 16.1%, 28.9% and 46.2%, respectively. Under LT and WL stress, all root indexes of snapdragon were significantly reduced, and inoculation with AMF (LTI and WLI) could effectively alleviate the inhibition effect of LT or WL stress on root development of snapdragon. Under LT stress, the total root length, total root surface area, total root projection area, total root volume, average root diameter, root tip number, and root tip bifurcation number increased by 21.4%, 34.5%, 27.1%, 35.3%, 13.8%, 42.5% and 35.2%, respectively, in the AMF (LTI) group. Compared with WL, the root indexes of WLI increased by 37.7%, 72.8%, 63.9%, 43.6%, 18.5%, 22.8% and 62.7%, respectively. And the total root length, root tip number, and root tip bifurcation number of LTWLI group were increased by 37.3%, 29.9% and 40.2% than that in LTWL group, respectively (**Supplementary Figure S2**; **Table 1**).

**Table 1:** Effects of AMF on root parameter of snapdragon under low temperature and/or weak light stresses.

Treatments	Total length of root (cm)	Total surface area of root (cm^2^)	Total projected area of root (cm^2^)	Total volume of root (cm3)	Average diameter of root (mm)	Number of root tips	Number of root branches
NTNL	1430.56 ± 36.69b	153.71 ± 5.41b	47.65 ± 1.72b	1.26 ± 0.06b	0.31 ± 0.01bc	6704 ± 29b	6631 ± 119c
NTNLI	1916.15 ± 15.91a	202.74 ± 6.88a	65.86 ± 2.56a	1.63 ± 0.05a	0.36 ± 0.01a	8640 ± 73a	9694 ± 96a
LT	1049.79 ± 38.34d	94.31 ± 5.48d	33.92 ± 1.96d	0.68 ± 0.02d	0.29 ± 0.02bcd	3766 ± 45e	5148 ± 69d
LTI	1274.80 ± 56.16c	126.89 ± 5.54c	43.14 ± 1.48c	0.92 ± 0.03c	0.33 ± 0.01ab	5366 ± 56c	6958 ± 42b
WL	785.37 ± 21.49e	56.58 ± 2.66e	16.59 ± 0.68f	0.39 ± 0.02f	0.27 ± 0.03cd	3312 ± 113f	3142 ± 80e
WLI	1081.82 ± 79.99d	97.76 ± 6.49d	27.19 ± 1.05e	0.56 ± 0.03e	0.32 ± 0.01abc	4068 ± 47d	5111 ± 70d
LTWL	362.46 ± 11.05g	39.04 ± 1.64f	11.31 ± 0.53g	0.26 ± 0.01g	0.22 ± 0.01e	2121 ± 22h	1706 ± 36g
LTWLI	497.75 ± 17.43f	51.56 ± 1.65ef	13.77 ± 0.36fg	0.31 ± 0.03fg	0.25 ± 0.02de	2756 ± 31g	2391 ± 47f
Test of significance							
AMF	**	**	*	*	**	***	**
Low temperature	**	**	*	**	*	**	*
Weak light intensity	***	***	***	***	**	**	**
Low temperature×Weak light intensity	***	***	***	***	***	***	***
AMF×Low temperature	NS	NS	NS	*	NS	*	NS
AMF×Weak light intensity	**	**	**	**	NS	**	*
AMF×Low temperature and weak light intensity	***	***	***	***	***	***	***

All data in the tables were expressed as means ± standard error (n=3), and different lowercase letters indicate a significant difference among the means at p < 0.05 by Duncan’s multiple range tests. And p-values of two-way ANOVAs of AMF, low temperature, weak light intensity, Low temperature and weak light intensity and their interaction are indicated as p<0.05, *p<0.01, **p<0.001, ***; ns, not significant.

Significance test showed that AMF, LT, WL, LT×WL had significant effects on total root length, total root surface area, total root projected area, total root volume, average root diameter, root tip number, and root tip bifurcation number (*p*<0.05), while AMF×LT only had significant effects on total root volume and root tip number (*p*<0.05). AMF×WL had significant effects on root parameters other than the number of root tips (*p*<0.01), while LT×WL, AMF ×LT×WL had a very significant effect on all of root parameters (*p*<0.001) (**Supplementary Figure S2**; **Table 1**).

### 3.2 Physiological analysis

#### 3.2.1 Effects of AMF on cell membrane permeability and osmoregulatory substances of snapdragon under low temperature and/or weak light stress

Under NTNL condition, inoculation with AMF (NTNLI) significantly increased the soluble sugar and soluble protein content by 20.3% and 22.1%, respectively (**Table 2**). Under LT stress, inoculation with AMF (LTI) could effectively reduce the relative conductivity of plants, and increase the contents of soluble sugar and soluble protein. Compared with LT, the relative conductivity of LTI group significantly decreased by 19.9%, while soluble sugar and soluble protein significantly increased by 12.3% and 12.6%, respectively. Compared with WL, inoculation with AMF (WLI) could effectively increase soluble sugar content by 20.8%. Compared with LTWL group, the relative conductivity was significantly decreased by 13.3%, while soluble sugar, soluble protein and proline were increased by 43.8%, 30.9% and 22.1%, respectively, more than that in LTWLI group (**Table 2**).

**Table 2:** Effects of AMF on cell membrane permeability and osmoregulation substance of snapdragon under low temperature and/or weak light stresses.

Treatments	Relative conductivity (%)	MDA (μmol·g^-1^)	Soluble sugar (mg·g^-1^)	Soluble protein (mg·g^-1^)	Proline (μg·g^-1^)
NTNL	15.72 ± 0.25d	0.011 ± 0.001d	11.25 ± 0.38d	21.51 ± 0.53d	39.32 ± 4.14ab
NTNLI	14.12 ± 0.43d	0.008 ± 0.001d	13.53 ± 0.35c	26.26 ± 1.36c	42.45 ± 0.56a
LT	31.34 ± 0.89b	0.023 ± 0.001b	15.84 ± 0.34b	32.84 ± 1.26b	31.72 ± 0.73cd
LTI	26.12 ± 0.42c	0.021 ± 0.001bc	18.07 ± 0.57a	37.58 ± 0.72a	34.83 ± 0.55bc
WL	26.36 ± 0.50c	0.021 ± 0.001bc	7.83 ± 0.16f	17.01 ± 0.66ef	27.28 ± 0.63de
WLI	23.89 ± 0.13c	0.018 ± 0.001c	9.89 ± 0.29e	19.14 ± 0.62de	30.42 ± 1.14cde
LTWL	37.35 ± 0.39a	0.031 ± 0.002a	4.15 ± 0.22g	10.86 ± 0.55g	20.77 ± 1.09f
LTWLI	32.95 ± 2.07b	0.028 ± 0.001a	7.39 ± 0.29f	15.72 ± 0.64f	26.65 ± 0.39e
Test of significance					
AMF	NS	*	*	*	*
Low temperature	***	**	**	**	**
Weak light intensity	**	**	**	**	**
Low temperature×Weak light intensity	***	***	***	***	***
AMF×Low temperature	**	**	***	***	**
AMF×Weak light intensity	**	**	*	*	**
AMF×Low temperature and weak light intensity	***	***	**	**	**

All data in the tables were expressed as means ± standard error (n=3), and different lowercase letters indicate a significant difference among the means at p < 0.05 by Duncan’s multiple range tests. And p-values of two-way ANOVAs of AMF, low temperature, weak light intensity, Low temperature and weak light intensity and their interaction are indicated as p<0.05, *p<0.01, **p<0.001, ***; ns, not significant.

Significance test showed that LT, WL, LT×WL, AMF×LT, AMF×WL, and AMF×LT×WL had significant effects on relative conductivity, MDA, soluble sugar and proline of snapdragon (*p*<0.05) (*p*<0.05), while AMF only had significant effects on MDA, soluble sugar and proline of snapdragon (*p*<0.05) (**Table 2**).

#### 3.2.2 Effects of AMF on snapdragon enzyme activities under low temperature and/or weak light stress

Compared with NTNL group, SOD, POD, CAT, APX, DHAR, MDHAR and GR of NTNLI groups increased significantly by 11.9%, 4.5%, 9.1%, 0.8%, 1.5%, 2.9% and 2.8%, respectively. The activities of SOD, POD, CAT, APX, DHAR, MDHAR and GR were significantly decreased under LT, WL and LTWL stress, but inoculation with AMF (LTI, WLI and LTWLI) could effectively improve the activities of these enzymes. Compared with LT, SOD, POD, CAT, APX, DHAR, MDHAR and GR in LTI group were significantly increased by 10.9%, 33.4%, 10.2%, 11.7%, 12.0%, 20.8% and 7.2%, respectively. SOD, POD, CAT, APX, DHAR, MDHAR and GR in WLI group were significantly higher than those in WL group by 12.2%, 25.2%, 11.2%, 26.7%, 22.1%, 28.7% and 27.3%, respectively. Compared with LTWL group, SOD, POD, CAT, APX, DHAR, MDHAR and GR of LTWLI groups increased significantly by 32.6%, 23.7%, 22.0%, 37.2%, 22.7%, 46.3% and 32.3%, respectively (**Table 3**).

**Table 3:** Effects of AMF on antioxidant enzyme activity of snapdragon under low temperature and/or weak light stresses.

Treatments	SOD (U/g·min)	POD (U/g·min)	CAT (U/g·min)	APX μ/(min·mg)	DHAR μ/(min·mg)	MDHAR μ/(min·mg)	GR μ/(min·mg)
NTNL	50.38 ± 1.34b	221.04 ± 9.91a	100.22 ± 2.55b	20.42 ± 0.69a	60.79 ± 2.14a	15.87 ± 0.83a	95.97 ± 1.77a
NTNLI	56.33 ± 1.06a	231.03 ± 3.61a	109.33 ± 4.85a	20.58 ± 0.52a	61.68 ± 1.63a	16.33 ± 0.19a	98.62 ± 2.08a
LT	37.52 ± 0.49d	137.11 ± 8.51c	80.47 ± 1.21d	15.32 ± 0.26c	45.48 ± 2.31c	10.73 ± 0.75c	72.65 ± 1.72c
LTI	41.62 ± 0.63c	182.88 ± 7.37b	88.69 ± 1.16c	17.11 ± 0.34b	50.95 ± 0.55b	12.96 ± 0.53b	77.88 ± 0.74b
WL	34.82 ± 0.85e	109.01 ± 5.78d	64.15 ± 1.46f	12.09 ± 0.61d	39.26 ± 0.87d	7.91 ± 0.18e	62.60 ± 1.15d
WLI	39.07 ± 0.21d	136.51 ± 10.57c	71.32 ± 1.11e	15.32 ± 0.53c	47.95 ± 1.86bc	10.18 ± 0.11cd	79.66 ± 1.75b
LTWL	21.65 ± 0.52g	82.71 ± 2.21e	45.74 ± 1.74h	7.67 ± 0.46f	31.92 ± 0.87e	5.94 ± 0.29f	42.46 ± 1.39f
LTWLI	28.71 ± 1.01f	102.29 ± 5.53d	55.75 ± 0.91g	10.52 ± 0.34e	39.16 ± 1.33d	8.69 ± 0.55de	56.18 ± 1.17e
Test of significance							
AMF	*	*	*	NS	NS	NS	NS
Low temperature	**	**	**	**	**	**	*
Weak light intensity	**	***	***	**	**	**	**
Low temperature×Weak light intensity	***	***	***	***	***	***	***
AMF×Low temperature	*	*	*	*	*	*	*
AMF×Weak light intensity	*	**	**	**	*	**	*
AMF×Low temperature and weak light intensity	**	**	***	**	**	**	**

All data in the tables were expressed as means ± standard error (n=3), and different lowercase letters indicate a significant difference among the means at p < 0.05 by Duncan’s multiple range tests. And p-values of two-way ANOVAs of AMF, low temperature, weak light intensity, Low temperature and weak light intensity and their interaction are indicated as p<0.05, *p<0.01, **p<0.001, ***; ns, not significant.

Significance test showed that LT, WL, LT×WL, AMF×LT, AMF×WL and AMF×LT×WL had significant effects on SOD, POD, CAT, APX, GR and MDHAR activities of snapdragon (*p*<0.05), while AMF only had significant effects on SOD, POD and CAT (*p*<0.05) (**Table 3**).

#### 3.2.3 Effects of AMF on antioxidant content in the ASA-GSH cycle of snapdragon under low temperature and/or weak light stress

Under NTNL, inoculation with AMF (NTNLI) increased ASA and GSH content, ASA/DHA (oxidized ascorbate) and GSH/GSSG (oxidized glutathione) by 10.0%, 22.1%, 3.1% and 31.0%, respectively. Under LT, WL and LTWL stress, Under LTWL stress, the contents of ASA and GSH in AMF group increased significantly, as did the ratios of ASA/DHA and GSH/GSSG. Among them, under LT, inoculation with AMF (LTI) increased ASA and GSH content, ASA/DHA, GSH/GSSG by 10.9%, 12.1%, 40.3% and 22.0%, respectively. Compared with WL, ASA content, GSH content, ASA/DHA, GSH/GSSG in WLI group increased by 14.7%, 12.8%, 42.3% and 22.7%, respectively. ASA content, GSH content, ASA/DHA and GSH/GSSG in LTWLI group were increased by 9.8%, 11.1%, 13.8% and 26.0%, respectively than that in LTWL group (**Table 4**).

**Table 4:** Effects of AMF on antioxidants in ASA-GSH cycle of snapdragon under low temperature and/or weak light stresses.

Treatments	ASAcontent nmol·g^-1^	GSHcontent nmol·g^-1^	ASA/DHA	GSH/GSSG
NTNL	777.96 ± 35.25f	127.25 ± 5.19f	6.37 ± 0.18f	2.26 ± 0.07g
NTNLI	855.18 ± 21.09f	155.32 ± 6.14e	6.57 ± 0.15f	2.95 ± 0.16f
LT	1132.14 ± 14.36e	176.26 ± 10.95de	7.69 ± 0.28e	3.42 ± 0.14e
LTI	1255.28 ± 35.42d	197.62 ± 3.93c	10.79 ± 0.48c	4.17 ± 0.06d
WL	1228.24 ± 52.56de	204.68 ± 7.76c	9.54 ± 0.24d	4.22 ± 0.12d
WLI	1408.97 ± 11.66c	230.98 ± 2.55b	13.58 ± 0.28b	5.18 ± 0.09c
LTWL	1586.23 ± 40.32b	253.37 ± 13.69b	13.36 ± 0.31b	6.69 ± 0.08b
LTWLI	1741.65 ± 52.09a	281.61 ± 11.38a	15.21 ± 0.54a	8.43 ± 0.15a
Test of significance				
AMF	NS	*	NS	*
Low temperature	**	**	*	*
Weak light intensity	**	**	**	**
Low temperature×Weak light intensity	***	***	***	***
AMF×Low temperature	**	**	**	**
AMF×Weak light intensity	***	***	***	***
AMF×Low temperature and Weak light intensity	***	***	***	***

All data in the tables were expressed as means ± standard error (n=3), and different lowercase letters indicate a significant difference among the means at p < 0.05 by Duncan’s multiple range tests. And p-values of two-way ANOVAs of AMF, low temperature, weak light intensity, Low temperature and weak light intensity and their interaction are indicated as p<0.05, *p<0.01, **p<0.001, ***; ns, not significant.

Significance test showed that LT, WL, and AMF×LT had significant effects on GSH and ASA content, GSH/GSSG, and ASA/DHA in snapdragon (*p*<0.05). However, AMF only had significant effects on GSH content and GSH/GSSG (*p*<0.05), and LT×WL, AMF×WL and AMF×LT×WL had significant effects on the above indexes (*p*< 0.001) (**Table 4**).

### 3.3 Transcriptional analysis

#### 3.3.1 Overview of RNA-seq data

**Supplementary Table S1** shows the sequencing profiles of 24 cDNA libraries. The raw base data of the samples ranged from 5.56 to 7.81 G and the cleaned base data ranged from 5.16 to 6.89 G per sample, which agreed with the expectations based on the RNA-seq library construction. The average effective rate of reads was 88.95% and the contents of the Q20 and Q30 bases were no less than 99.99% and 98.85%, respectively. The average GC value was about 45.29%, which in combination with the above results, showed that the transcriptome data of each sample met the requirements for further information analysis (**Supplementary Table S1**). In addition, based on the snapdragon genome database (http://bioinfo.sibs.ac.cn/Am/), a total of 8 chromosomes, 37,232 genes, and 51,724 transcripts were found in the snapdragon libraries. From the GO and KEGG databases, 26,424 and 10,723 genes were annotated, respectively (**Supplementary Table S2**). HISAT was used to compare the pre-processed valid data with the reference genome of snapdragon, and showed that the proportion of clean reads that were matched with a corresponding gene was > 84%. The mapped reads from the eight treatment groups accounted for at least 67.14% of the total filtered data, while the percentage of sequenced sequences with multiple alignment positions on the reference sequence was ≥ 10.74%, with a relatively high comparison rate. These mapped reads were used for subsequent analysis (**Supplementary Table S3**).

#### 3.3.2 Analysis of differentially expressed genes

**Figure 1:**
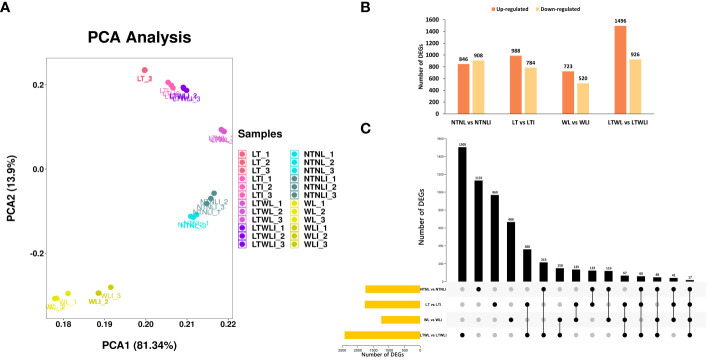
Differentially expressed genes (DEGs) in the seedlings of snapdragon inoculated with AMF and non-inoculation under low temperature (LT) and/or weak light (WL) stress. **(A)** Principal Component Analysis (PCA) of transcriptomes (treatments and replicates), **(B)** Statistics on the number of up- and down-regulated DEGs, **(C)** UpSet diagram of DEGs in different comparison group.

To screen the differentially expressed genes (DEGs) of mycorrhizal snapdragon respond to low LT, WL, and LTWL stress, we set up four comparison groups: NTNL vs NTNLI, LT vs LTI, WL vs WLI, and LTWL vs LTWLI. The RNA-seq results showed that the first principal component (PC1) explained 81.34% of the variance, and the second principal component (PC2) explained 13.9% of the variance, with a cumulative contribution rate of 95.24%. The principal component comparison of each group of samples was relatively aggregated, and there was more separation between the LTWL vs LTWLI than the other comparison groups (NTNL vs NTNLI, LT vs LTI, WL vs WLI) (**Figure 1A**). The differential gene screening conditions were *p* < 0.05, |log_2_ (Foldchange)|>1. The study found that LTWL vs LTWLI had the largest number of DEGs, with 2422, of which 1496 genes were up-regulated and 926 genes were down-regulated. The NTNL vs NTNLI comparison group had 846 up-regulated and 908 down-regulated genes. In LT vs LTI there were 988 up-regulated and 784 down-regulated genes, while in WL vs WLI there were 723 up-regulated and 520 down-regulated genes (**Figure 1B**). Upset diagram showed that, of all the 5606 DEGs in the four comparison groups, 1131 (20.2%) were expressed in NTNL vs NTNLI, 969 (17.3%) were expressed in LT vs LTI, 666 (11.9%) were expressed in WL vs WLI, and 1505 (26.8%) were expressed in LTWL vs LTWLI. There are 360, 150, and 135 genes in common between LT vs LTI and LTWL vs LTWLI, WL vs WLI and LTWL vs LTWLI, LT vs LTI and WL vs WLI, respectively. And 67 genes (26.8%) were shared among the LT vs LTI, WL vs WLI, and LTWL vs LTWLI comparison groups, while only 17 DEGs were expressed in all four comparison groups (**Figure 1C**).

**Figure 2:**
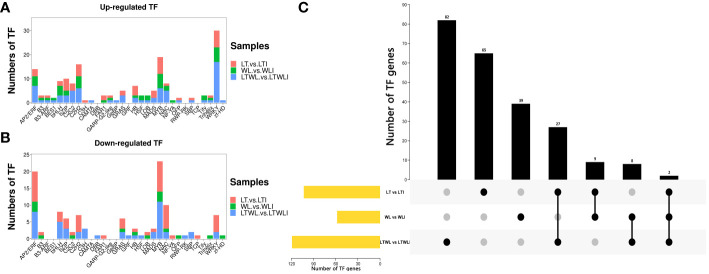
Differentially expressed transcription factors (TFs) in the seedlings of snapdragon inoculated with AMF and non-inoculation under low temperature (LT) and/or weak light (WL) stress. **(A)** Statistics of up-regulated TFs, **(B)** Statistics of down-regulated TFs, and **(C)** UpSet diagram of TFs in different comparison group.

Transcription factors (TFs) are involved in regulating the expression of downstream target gene and play important roles in the stress responses of plants. In this experiment, a total of 233 TFs were identified from the LT vs LTI, WL vs WLI, and LTWL vs LTWLI comparison groups, covering 22 TF families (**Supplementary Table S4**; **Figure 2**). A total of 119 TFs were identified in the LTWL vs LTWLI comparison group (far more than the 103, 58 TFs in the LT vs LTI and WL vs WLI groups). Of the TFs in the LT vs LTI, WL vs WLI, and LTWL vs LTWLI groups, 51, 43, and 72 members were up-regulated, while 52, 15, and 47 members were down-regulated (**Figures 2A, B**). The up-regulated TFs were concentrated in the WRKY and MYB families, while the down-regulated TFs were concentrated in the MYB and AP2/ERF families. In addition, 65, 39, and 82 TFs were expressed exclusively in the LT vs LTI, WL vs WLI, and LTWL vs LTWLI groups, respectively (**Figure 2C**), with the TFs of the three comparison groups focused in the AP2/ERF, bHLH, MYB, NAC, and WRKY families. Therefore, the AP2/ERF, bHLH, MYB, NAC, and WRKY family genes were selected for qRT-PCR analysis (**Supplementary Table S7**).

**Figure 3:**
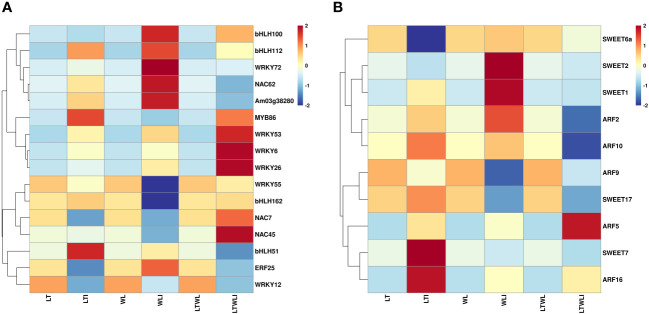
Heatmaps representing the qRT-PCR analysis of expression levels of **(A)** transcription factors and **(B)** the auxin response factor (ARF) family of genes and sugars will eventually be exported transporter (SWEET) family of genes in the various comparison groups. Relative expression levels of individual genes among treatments are indicated with varying colors, red represents upregulation, blue represents downregulation, samples and genes are represented by different columns and rows, respectively.

The qRT-PCR showed that the expression patterns of TFs in the different comparison pairs fell into two clusters, one with bHLH100, bHLH112, WRKY72, NAC62, Am03g38280, MYB86, WRKY6, WRKY26, and WRKY53 and one with WRKY55, bHLH162, NAC7, NAC45, bHLH51, ERF25, and WRKY12 (**Figure 3A**; **Supplementary Table S8**). Under LT, AMF induced up-regulated expression of most transcription factor genes, except for bHLH100, WRKY55, NAC7, NAC45, ERF25, and WRKY12. Under WL, AMF induced up-regulated expression of *bHLH100, bHLH112, WRKY72, NAC62, Am03g38280, MYB86, WRKY53, WRKY6, WRKY26*, and *bHLH51*. And AMF induced up-regulated expression of the *bHLH100, bHLH112, WRKY72, MYB86, WRKY53, WRKY6, WRKY26*, and *NAC45* under LTWL stress. Among them, *bHLH112, WRKY72, MYB86, WRKY53, WRKY6*, and *WRKY26* were up-regulated after AMF inoculation under three stress conditions (**Figure 3A**).

In addition, the expression analysis of auxin response factor (ARF) family genes and sugars will eventually be exported transporter (SWEET) family genes in the various comparison groups showed that the expression patterns of these two families of genes could also be clustered into 2 groups, SWEET6a, SWEET2, SWEET1, ARF2, and ARF10 clustered into one group, and ARF9 SWEET17, ARF5, SWEET7, ARF16 clustered into another group. The *ARF5* and *ARF16* were up-regulated when plants under LT, WL, and LTWL conditions were inoculated with AMF. *SWEET1, ARF2*, and *ARF10* were up-regulated by AMF inoculation only under LT and WL stress. Furthermore, AMF inoculation only induced up-regulated expression of *SWEET2/6a* under WL stress, and *SWEET17/7* under LT stress (**Figure 3B**; **Supplementary Table S8**).

#### 3.3.3 GO function analysis of differentially expressed genes

**Figure 4:**
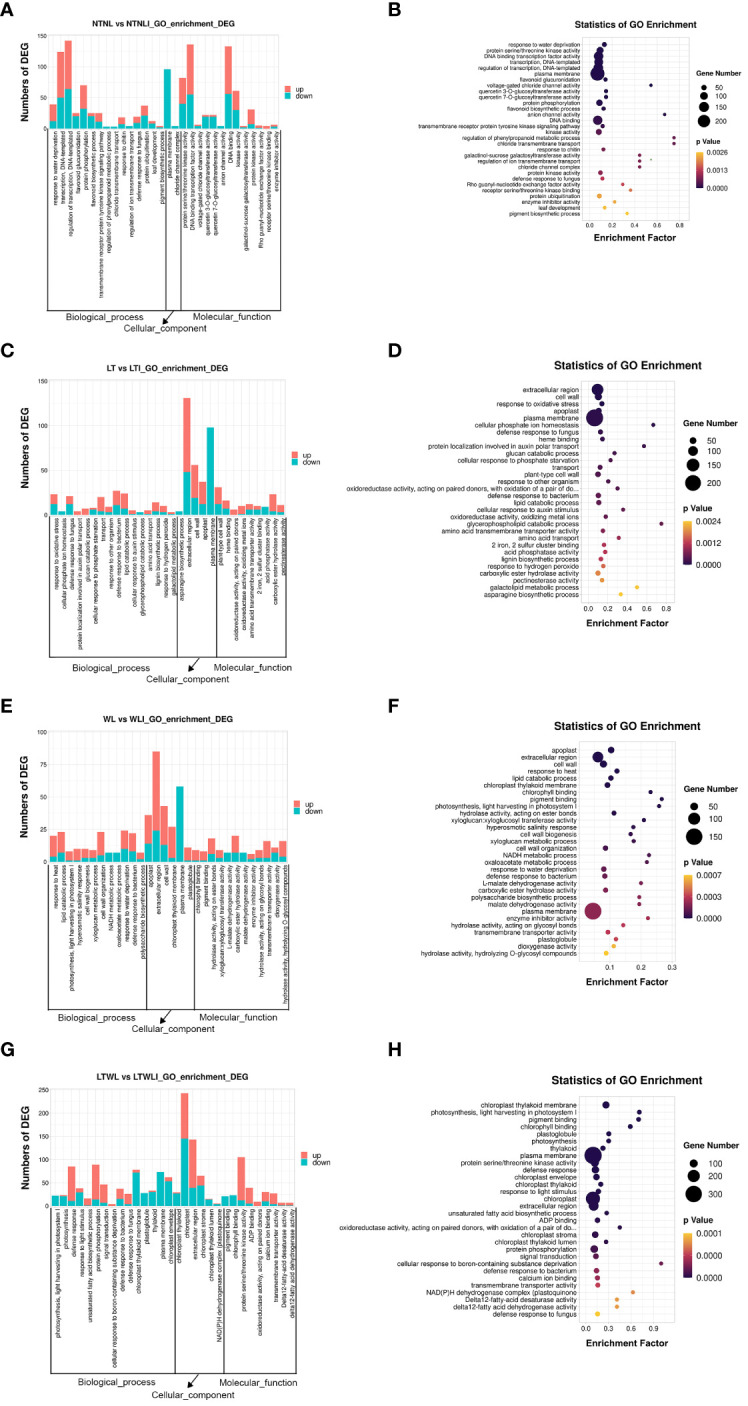
Functional categorization of DEGs based on gene ontology (GO) in the seedlings of snapdragon inoculated with AMF and non-inoculation under low temperature (LT) and/or weak light (WL) stress. **(A, B)** NTNL_vs._NTNLI, **(C, D)** LT_vs._LTI, **(E, F)** WL_vs._WLI, **(G, H)** LTWL_vs._LTWLI.

The GO terms enriched in DEGs sets were identified. Accumulation and bubble diagrams were used to represent the top 30 GO items in biological processes (BP), cellular components (CC), and molecular functions (MF). In NTNL vs NTNLI, a total of 1382 DEGs were mapped to the GO database, accounting for 74.10% of all DEGs in that comparison group (**Figures 4A, B**). In BP, DEGs were mainly involved in response to water deprivation (GO: 0009414), transcription (GO: 0006351), and regulation of transcription (GO: 0006355), with 27, 74, and 78 up-regulated DEGs and 12, 50, and 64 down-regulated DEGs in each category, respectively. In CC, the DEGs were mainly involved in pigment biosynthetic processes (GO: 0046148) and the plasma membrane (GO: 0005886), with 2 and 138 up-regulated DEGs and 2 and 96 down-regulated DEGs in the two categories, respectively. In MF, DEGs were mainly involved in protein serine/threonine kinase (GO: 0004674), DNA binding transcription factor activity (GO: 0003700), and voltage-gated chloride channel activity (GO: 0005247), with 42, 81, and 2 up-regulated DEGs and 40, 55, and 4 down-regulated DEGs in each category, respectively. In conclusion, under non-stressful conditions, AMF inoculation induced more up-regulated DEGs, and most DEGs were involved in basic biological processes and cellular component synthesis (**Figures 4A, B**).

A total of 1345 DEGs in the LT vs LTI group were mapped to the GO database, accounting for 78.61% of the group’s total DEGs (**Figures 4C, D**). In BP, DEGs were mainly involved in the response to oxidative stress (GO: 0006979), cellular phosphate ion homeostasis (GO: 0030643), and defense response to fungus (GO: 0050832), with 11, 0, and 9 up-regulated DEGs and 12, 4, and 12 down-regulated DEGs in each category, respectively. In CC, DEGs were mainly involved in the asparagine biosynthetic process (GO: 0006529), extracellular region (GO: 0005576), and cell wall (GO: 0005618), with 83, 37 and 25 up-regulated DEGs and 48, 19, and 12 down-regulated DEGs in the different categories, respectively. In MF, DEGs were involved in heme binding (GO:0020037) and oxidoreductase activity (GO: 0016717, GO: 0016722), with 9, 6, and 9 up-regulated DEGs and 7, 0, and 1 down-regulated DEGs, respectively. These results showed, that under LT stress, the DEGs of AMF-inoculated snapdragon were mostly involved in oxidative stress, defense response, phosphorus metabolism, and photosynthetic pigment synthesis of plants (**Figures 4C, D**).

A total of 919 DEGs in WL vs WLI group were mapped to the GO database, accounting for 70.75% of the group’s total DEGs (**Figures 4E, F**). In BP, DEGs were mainly involved in the response to heat (GO: 0009408), lipid catabolic process (GO: 0016042), and photosynthesis in light harvesting photosystem I (GO: 0009768), with 16, 16, and 7 up-regulated DEGs and 4, 7 and 1 down-regulated DEGs in each category, respectively. In CC, DEGs were mainly involved in the apoplast (GO: 0048046), extracellular region (GO: 0005576), and cell wall (GO: 0005618), with 22, 61, and 30 up-regulated DEG and 14, 24, and 13 down-regulated DEGs, respectively. In MF, DEGs mainly participated in the chlorophyll binding (GO: 0016168), pigment binding (GO: 0031409), and hydrolase activity acting on ester bonds (GO: 0016788), with 8, 7, and 12 up-regulated DEGs and 1, 1, and 6 down-regulated DEGs in the different categories, respectively. It could be seen that, under WL stress, AMF inoculation induced more up-regulation of gene expression and the DEGs were mostly involved in plant thermal stimulation response, lipid catabolism, light capture reaction in photosynthesis, chlorophyll binding, and other processes (**Figures 4E, F**).

The LTWL vs LTWLI group had a total of 1834 DEGs mapped to the GO database, accounting for 70.40% of the group’s total DEGs (**Figures 4G, H**). In BP, The DEGs were mainly involved in the photosynthesis light harvesting photosystem I (GO: 0009768), photosynthesis (GO: 0015979), and defense response (GO: 0006952), with 0, 2, and 76 up-regulated DEGs and 22, 21, and 11 down-regulated DEGs, respectively. In CC, DEGs were mainly involved in the chloroplast thylakoid membrane (GO: 0009535), plastoglobule (GO: 0010287), and thylakoid (GO: 0009579), with 6, 0, and 3 up-regulated DEGs and 72, 28, and 30 down-regulated DEGs, respectively. In MF, DEGs participated mainly in pigment binding (GO: 0031409), in leaves binding (GO: 0016168), and in protein serine/threonine kinase activity (GO: 0004674), with 0, 0, and 93 up-regulated DEGs and 21, 23, and 12 down-regulated DEGs, respectively. It could be seen that, under combined low temperature and low light stress, the DEGs of AMF-vaccinated snapdragon mainly participated in the light capture reaction, defense response, chloroplast structural components, and chlorophyll binding processes of plant photosynthesis (**Figures 4G, H**).

#### 3.3.4 KEGG metabolic pathway analysis

**Figure 5:**
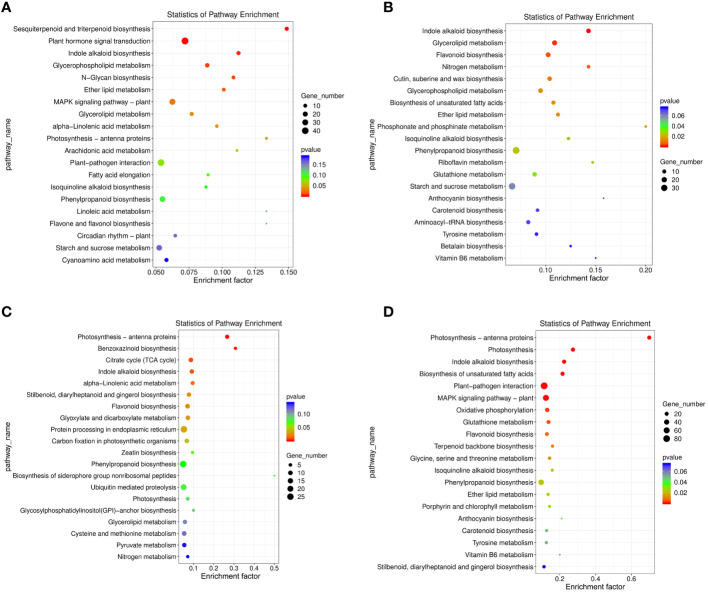
The KEGG metabolism pathway categories of the top 20 enriched pathways in the seedlings of snapdragon inoculated with AMF and non-inoculation under low temperature (LT) and/or weak light (WL) stress. **(A)** NTNL_vs._NTNLI, **(B)** LT_vs._LTI, **(C)** WL_vs._WLI, and **(D)** LTWL_vs._LTWLI.

KEGG annotation was also used for metabolic pathway enrichment analysis in the DEG sets found in the 4 comparison groups, using a corrected *p*-value < 0.05 as enrichment threshold. The top 20 enriched metabolic pathways were selected for further analysis and discussion (**Figure 5**; **Supplementary Table S5**). In the NTNL vs NTNLI group, the sesquiterpenoid and triterpenoid biosynthesis (ko00909), plant hormone signal transduction (ko04075), and indole alkaloid biosynthesis (ko00901) metabolic pathways were significantly enriched by 11, 45, and 11 DEGs, respectively (**Figure 5A**). In the LT vs LTI group, the indole alkaloid biosynthesis (ko00901), glycerolipid metabolism (ko00561), and flavonoid biosynthesis (ko00941) metabolic pathways were significantly enriched by 14, 17, and 16 DEGs, respectively (**Figure 5B**). In the WL vs WLI group, the photosynthesis-antenna proteins (ko00196), benzoxazinoid biosynthesis (ko00402), and citrate cycle (ko00020) pathways showed significant enrichment by 8, 4, and 10 DEGs, respectively (**Figure 5C**). In the LTWL vs LTWLI group, the photosynthesis-antenna proteins (ko00196), photosynthesis (ko00195), and indole alkaloid biosynthesis (ko00901) metabolic pathways were enriched by 21, 23, and 22 DEGs, respectively (**Figure 5D**). It could be seen that, under non-stressful conditions, the main metabolic pathways enriched in AMF-inoculated snapdragon were secondary metabolite synthesis, hormone signal transduction, and auxin substance synthesis. However, under LT or WL stress, AMF-inoculated snapdragon exhibited enrichment in a very different set of metabolic pathways, with the former enriched in pathways involved in plant hormone synthesis, lipid metabolism, and flavonoid metabolism, and the latter showing enrichment in pathways involved in synthesis of photosynthetic antenna protein and the synthesis of benzoxazinoids (BXs), which are important secondary metabolites with defensive and allelopathic effects. In addition, under LTWL, the enriched pathways in the AMF-inoculated snapdragon were mainly involved in metabolic pathways such as photosynthesis and plant hormone synthesis.

#### 3.3.5 DEGs involved in photosynthesis

Under LT, WL, or LTWL stress, 70% of DEGs induced by AMF inoculation were successfully annotated in the GO database, and these DEGs were mainly involved in oxidative stress, defense response, and photosynthesis. The KEGG metabolic pathway analysis showed that DEGs were also primarily involved in the photosynthesis, hormone signal transduction, and defense response secondary metabolite synthesis pathways. Therefore, we further focused on DEGs involved in photosynthesis and active oxygen metabolism (**Supplementary Table S9**).

**Figure 6:**
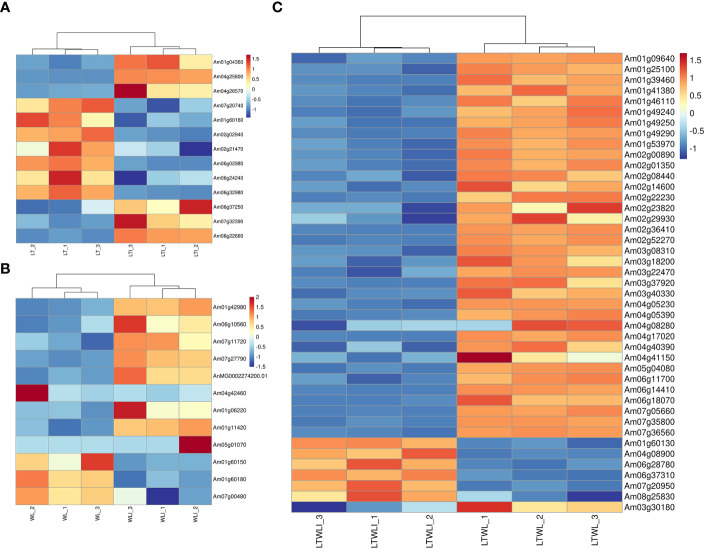
DEGs involved in photosynthesis and active oxygen metabolism of snapdragon inoculated with AMF and/or non-inoculation under low temperature (LT) and weak light (WL) stress. **(A)** LT_vs._LTI, **(B)** WL_vs._WLI, and **(C)** LTWL_vs._LTWLI.

There were four DEGs involved in photosynthesis in the LTI vs LT comparison group (**Figure 6A**): two DEGs encoding ribulose bisphosphate carboxylase small chains (Am04g25800, Am04g26570) and one DEG encoding Chlorophyllide-a oxygenase (Am01g04360) were up-regulated, and one gene, encoding light-harvesting complex-like protein 3 isotype 1(Am07g20740), was down-regulated.

In the WLI vs WL group, 6 DEGs were related to photosynthesis (**Figure 6B**): five were up-regulated, encoding photosystem II 22 kDa protein (Am01g42980), phototropic-responsive NPH3 family protein (Am06g10560), chlorophyll a-b binding protein of LHCII type 1-like(Am07g11720), thylakoid lumenal 17.9 kDa protein (Am07g27790), and photosystem II Q (b) protein D1(AnMG0002274200.01), and one was down-regulated, encoding photosynthetic NDH subunit of subcomplex B5 (Am04g42460).

There were 37 DEGs involved in photosynthesis in the LTWLI vs LTWL group, all of which were down-regulated (**Figure 6C**). These included four genes encoding photosystem I related proteins (Am01g39460, Am01g46110, Am02g01350, Am02g00890) and six genes encoding photosystem II related proteins (Am01g53970, Am02g08440, Am02g29930, Am02g52270, Am03g08310, Am04g40390), 12 genes encoding chlorophyll a/b binding protein (Am01g41380, Am01g46110, Am01g49240, Am01g49250, Am01g49290, Am03g18200, Am03g40330, Am04g05230, Am04g05390, Am04g08280, Am06g14410, and Am07g36560), and three encoding photosynthetic NDH subunit (Am02g22230, Am03g37920, Am06g18070).

#### 3.3.6 DEGs involved in ROS scavenging

The DEGs related to ROS scavenging were studied. There were 9 DEGs associated with active oxygen metabolism in the LTI vs LT comparison group (**Figure 6A**): three were up-regulated, namely L-ascorbate oxidase (Am06g37250), POD64 (Am07g32390) and transmembrane ascorbate ferrireductase 2 (Am08g22660); Six DEGs were down-regulated, including three iron/ascorbate family oxidoreductases (Am01g60160, Am02g02940, Am02g21470), POD16 (Am06g02980), nucleobase-ascorbate transporter 6 (Am06g24240) and Proline dehydrogenase 1 (Am06g32980).

In the WLI vs WL group, there were 6 DEGs involved in active oxygen metabolism (**Figure 6B**): three were up-regulated, namely L-ascorbate oxidase homolog (Am01g11420), nucleobase-ascorbate transporter 3 (Am05g01070), transmembrane ascorbate ferrireductase 3 (Am01g06220), and three genes were down-regulated, including two iron/ascorbate family oxidoreductases (Am01g60150, Am01g60180) and POD gene (Am07g00480).

There were 7 DEGs associated with active oxygen metabolism in the LTWLI vs LTWL group (**Figure 6C**): In the process of scavenging H2O2, four POD gene (Am04g08900, Am06g28780, Am06g37310, Am08g25830) were up-regulated, one DEG involving scavenging superoxide anion free radical (Fe-SOD, Am07g20950) and iron/ascorbate family oxidoreductase (Am01g60130) were up-regulated, one POD gene (Am03g30180) was down-regulated.

### 3.4 Validation of RNA-seq data by qRT-PCR

To verify the accuracy of the RNA-seq data, quantitative real-time PCR (qRT-PCR) was applied to 10, 9, and 17 DEGs from the LT vs LTI, WL vs WLI, and LTWL vs LTWLI groups, respectively (**Supplementary Tables S6, S7**). Thirty-seven pairs of fluorescently labeled quantitative primers were designed using the Primer 5.0 software, among which 36 pairs were DEG-specific primers and one was an internal reference gene primer (**Supplementary Table S7**). The main functions of the 36 verified DEGs were signal transduction, carbohydrate transport and metabolism, translation, and stress response (**Supplementary Figure S3**). Detailed information of each DEG can be found in **Supplementary Table S6**. These candidate genes showed positively correlations between their RNA-seq log_2_(Foldchange) and qRT-PCR log_2_(Foldchange) values in each treatment group (**Supplementary Figure S3**). The correlation R^2^ value was 86.48%, indicating that the measured expression levels of the 36 DEGs was in good agreement with the RNA-seq data (**Supplementary Figure S3**), which confirmed the relative rationality and accuracy of the RNA-seq analysis.

## 4 Discussion

**Figure 7:**
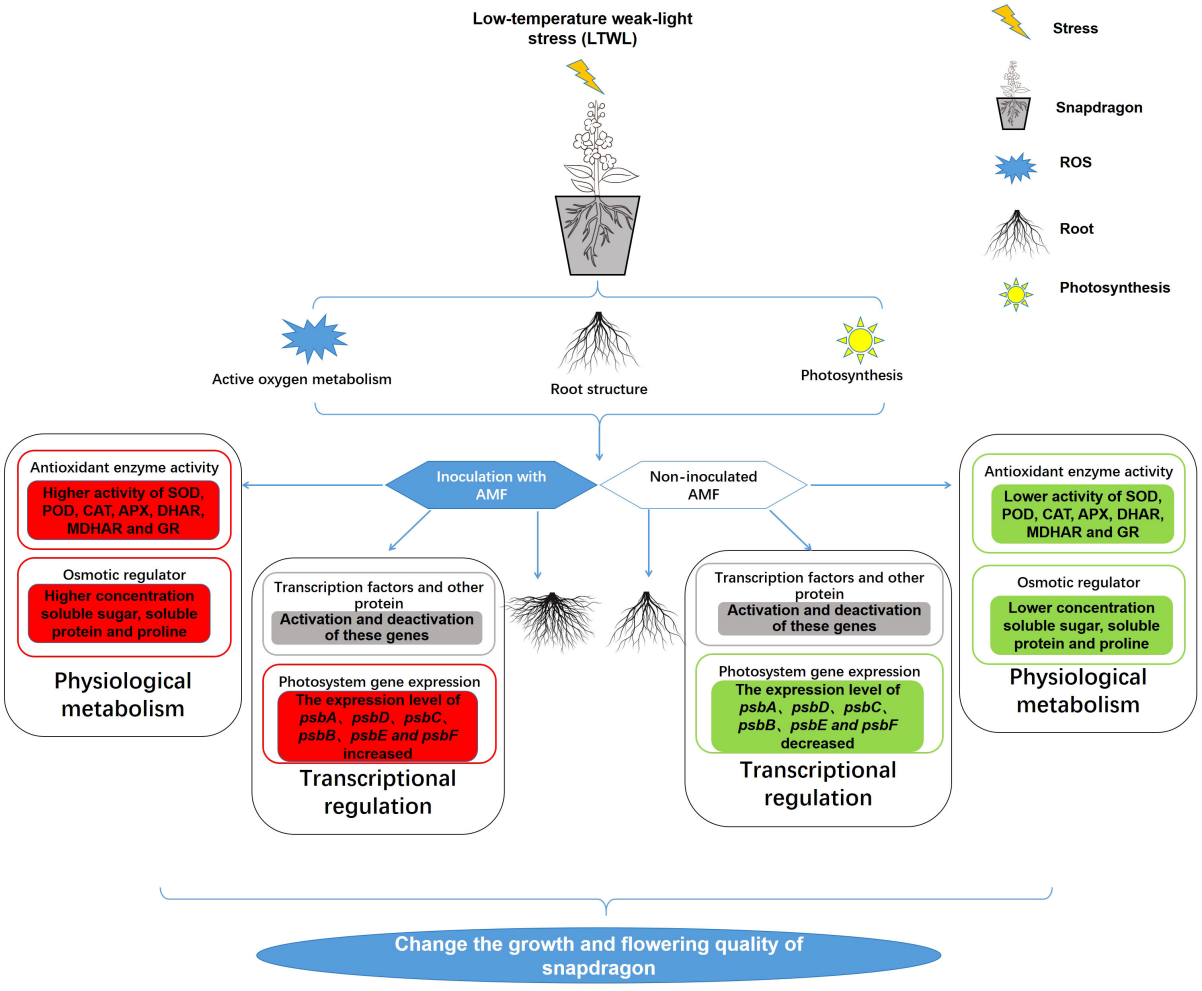
Summary figure of the effects of inoculation with AMF in the response of snapdragon to low temperature (LT) and/or weak light (WL) stress. The red squares in the figure represent the up-regulation of enzyme activity, substance quality or gene expression level, the green squares represent the down-regulation of enzyme activity, metabolite concentration, soluble protein levels or gene expression level, and the gray squares represent changes in gene expression levels.

Greenhouses represent an important agricultural production method globally, providing a suitable environment for plants grown in non-growing seasons (Costa and Heuvelink, 2004; Gong et al., 2021). In the winter cultivation of snapdragon, natural light and non-heated greenhouse facilities are common. However, these greenhouses often experience low temperature (LT) and weak light (WL) conditions, which can seriously affect the growth of seedlings and result in low flower quality. Studies have shown that AMF inoculation can alleviate plant response to LT or WL stress, but few reports have examined the transcriptomic response mechanisms by which AMF inoculated plants respond to LTWL stress. Therefore, this study focused on changes in physiological metabolism and the transcriptome of AMF-inoculated and non-inoculated snapdragon after 7 days exposure to LT, WL, and LTWL stress. The results have shown that LT, WL, and LTWL significantly inhibit the growth of snapdragon root system, the activity of antioxidant enzymes and the synthesis of osmotic regulator, but inoculation with AMF can mitigate these effects, combined with the results of transcriptomic sequencing, a series of differentially expressed genes (DEGs) were shown to be involved in photosynthesis, active oxygen metabolism, flavonoid synthesis, unsaturated fatty acid metabolism, and other pathways (**Figure 7**).

### 4.1 AMF inoculation promoted snapdragon root growth under low temperature and/or weak light conditions

When plants are exposed to adverse conditions, morphological changes can reflect the tolerance of plants to adversity, and the most direct response is the suppression of plant growth. Our previous study found that LT, WL, and LTWL stress obviously inhibited the growth of snapdragon, with the effect of LTWL stress being the most pronounced, but these effects were alleviated after inoculation with *F. mosseae* and *G. versiforme* (Li et al., 2023) (**Supplementary Figure S4**). This study focused on the effects of LT, WL and LT WL stress on the root system of snapdragon. Under the three kinds of stress, the root parameters of snapdragon were inhibited, and the plant root status could indicate the degree of stress, because LT limited the elongation and differentiation of root cells (Nagasuga et al., 2011; Zhu et al., 2015; Yamori et al., 2022), thereby reducing the biomass of roots. Similarly, WL stress decreased the perennial ryegrass root length, root surface area, root mass, and the root shoot ratios (Fu et al., 2020), which was basically consistent with the results of our study. However, there is also evidence that WL can promote the elongation of wheat roots, but reduce root branching (Page et al., 2012), indicating that WL has differentiated effects on different parts of plant roots, which may also depend on the type of plant roots. Previous studies have shown that AMF colonization can improve *Citrus reticulata* (Wu et al., 2016; Zhang et al., 2019a), *Hordeum vulgare* (Brown et al., 2013), and *Alnus glutinosa* (Orfanoudakis et al., 2010) root hair growth, enhance the vitality of plant roots, and promote the growth and development of plant roots and aboveground parts (Pavithra and Yapa, 2018), all these findings were consistent with the results of this study. In addition, we identified 19 DEGs associated with auxin synthesis, transport and response (**Figure 3B**), This suggests that AMF inoculation may be involved in the root formation of snapdragon by regulating auxin synthesis (Chen et al., 2017). However, it has also been reported that nutritional and reproductive traits of AMF are inhibited under light restriction conditions (Ballhorn et al., 2016). Therefore, whether the symbiotic system is beneficial to plants depends on the type of AMF inoculation, and is also related to the type and intensity of limiting factors. Plants cannot widely increase photosynthesis, and under such conditions, the costs of maintaining symbiotic relationships may outweigh the benefits in some conditions. Indeed, in some scenarios, symbiotic microorganisms can become parasitic microorganisms, resulting in reduced plant growth and reproduction (Lau et al., 2012).

### 4.2 Inoculation with AMF improved enzymatic and non-enzymatic antioxidant defense systems of snapdragon under low temperature and/or weak light conditions

Under LT, WL or combined stress, the photosynthetic rate of plants is reduced and growth and development are inhibited. In response to stress-induced oxidative damage, plants have evolved many enzymatic antioxidant systems to control the production and clearance of reactive oxygen species (ROS) (Kaya and Shabala, 2024). WL has previously been observed to cause ROS to accumulate, including hydrogen peroxide (H_2_O_2_) and superoxide anion (O_2_^·−^), which inhibits photosynthesis and slows plant growth (Fu et al., 2014).

In addition, compared with the single stress of LT or WL, the effect of LTWL was significantly greater than that of simple LT treatment, and the quantum yield of plants under the combined stress was higher, and the activities of superoxide dismutase, peroxidase and catalase were also higher (Xu et al., 2020). AMF colonization of sorghum (Liu et al., 2004) and cucumber (Liu et al., 2013) will also decrease, and WL also reduced the growth response of mycorrhiza (Konvalinková and Jansa, 2016). However, studies have found that under LT stress, AMF inoculation can activate the antioxidant defense system of *Elymus nutans* Griseb. and *Solanum melongena* (Chu et al., 2016; Pasbani et al., 2020), and AMF inoculation reduced the accumulation of H_2_O_2_ and O_2_^·−^ and increased the synthesis and activation of antioxidant enzymes in tall fescue under WL conditions (Hossain et al., 2015). In addition, AMF improved the growth of *Santalum album* experiencing shade stress (Binu et al., 2015). Similarly, in this study, under LT, WL, and LTWL conditions, the activities of SOD, POD, CAT, APX, DHAR and MDHAR were higher in AMF-inoculated snapdragon leaves and ROS were effectively cleared, which alleviated lipid peroxidation in cells and protected snapdragon from oxidative damage. This was also confirmed by GO enrichment analysis, which showed the differences between LT vs LTI, WL vs WLI, and LTWL vs LTWLI found that DEGs were mainly involved in ROS metabolic pathways, and the expression of genes such as POD were significantly up-regulated (**Table 3**). In summary, AMF inoculation activated the antioxidant oxidase scavenging system and effectively cleared the excessive ROS produced by LT, WL and LTWL stress. However, it was also found that, under cold stress, inoculation with *Diversispora versiformis* did not influence the activities of SOD, POD, and CAT in leaves and roots (Cao et al., 2022), which means that AMF may alleviate stress to plants by more than one pathway, and increases in antioxidant enzyme activity are just one of them. In addition, plants can also use non-enzymatic antioxidant defense system to protect themselves from oxidative damage under stress conditions, AsA and GSH and the ASA-GSH cycle are indispensable non-enzymatic antioxidants in plant organelles and cytoplasm and the main pathway of H_2_O_2_ clearance in plants. For example, under LT stress, the AsA and GSH levels in the leaves of AMF inoculated blueberry plants were higher than those in non-inoculated plants, but the levels of O_2_^·−^, H_2_O_2_, and MDA in the leaves were observed to be lower in AMF inoculated plants than non-inoculated plants (Zhang et al., 2013). The results of this study are consistent with those of this study. Under LT, WL and LTWL stress, the AsA and GSH contents in mycorrhizal snapdragon leaves were higher than those in unvaccinated plants. In conclusion, mycorrhizal snapdragon can protect plants from oxidative damage under LT, WL and LTWL stress by means of enzymatic and non-enzymatic antioxidant defense system.

### 4.3 AMF inoculation increased the content of osmotic regulatory substances in snapdragon under low temperature and/or weak light conditions

The accumulation of osmotic regulatory substances is a necessary mechanism for plants to cope with abiotic stress (Hassan et al., 2021). Among them, soluble sugars and soluble proteins are essential energy sources for plants, playing crucial roles in plant stress resistance. Environmental stress has been shown to affect the concentrations of soluble sugars and soluble proteins (Kobylińska et al., 2018; Hassan et al., 2021). In addition, proline is also indispensable in the characterization of abiotic stress, and the accumulation of proline is a common physiological response of plants to various stresses (Ozden et al., 2009). For example, the levels of electrical conductance, soluble proteins, and proline in pepper leaves decreased under LTWL conditions (Tang et al., 2021), and WL also leads to the decrease of soluble sugar and soluble protein contents of leguminous plants, and delayed flowering, thus affecting plant yield (Shukla et al., 2018). In this study, Under LTWL conditions, snapdragons also showed basically the same response, and AMF inoculation could alleviate the adverse effects of LTWL stress on plants. Higher concentrations of soluble sugars and starches were observed in AMF-inoculated barley at 15°C (Hajiboland et al., 2019), and AMF inoculation of eggplant was able to accumulate protective molecules and reduce the damage to cell membranes caused by LT (Pasbani et al., 2020). Compared with LTWL group, the concentrations of soluble sugar, proline and soluble protein in mycorrhizal snapdragons (LTWLI) in this study were also higher under the three stress conditions. Previous studies confirmed that mycorrhizal snapdragons had a higher photosynthetic rate (Li et al., 2023), which provided additional carbon skeleton for the synthesis of protective molecules such as carbohydrates and proline. In addition, the synthesis of more photosynthetic organic matter can better satisfy the resistance of the symbiotic system to LTWL. However, Abdel Latef and He (2011) reported that the proline concentration in mycorrhizal tomato plants under salt stress was lower than in non-mycorrhizal tomato plants, possibly due to the type and degree of stress and the tolerance of this species.

### 4.4 AMF changed the expression patterns of key genes in snapdragon under low temperature and/or weak light conditions

Many types of transcription factors play important regulatory roles in plant stress response. Xin et al. (2013) found that overexpression of WRKY, AP2/ERF, MYB, Homeobox, NAC and bHLH family genes participated in grape LT response and may enhance grape cold resistance. The overexpression of *Miscanthus lutarioriparius MlNAC5* in *Arabidopsis thaliana* significantly enhanced its cold tolerance through the transcriptional regulation of some stress response marker genes (Yang et al., 2015). Overexpression of soybean *GmERF9* enhanced drought and cold tolerance of transgenic tobacco (Zhai et al., 2017). Soitamo et al. (2003) showed that the combination of WL and LT enhanced the expression of several cold-responsive transcription factor genes encoding MYB, NAC, WRKY, and AP2/ERF proteins. AMF colonization of the root system of host plants can lead to the establishment of symbiotic relationships and cause changes in gene transcription (Bucher et al., 2014), It has been reported that AMF has multiple benefits on plant stress resistance by up-regulating or down-regulating the expression level of transcription factors. For example, under salt stress, WRKY, MYB, and ERF appeared to be the core regulatory components leading to acquired salt tolerance in mycorrhizal *Sesbania* (Ren et al., 2019). WRKY, NAC, and MYB were found to play key roles in the salt stress response of mycorrhizal durum wheat (*Triticum turgidum* L. subsp) (Puccio et al., 2023). In the later stages of symbiosis, AP2, ERF, Myb, WRKY, and bHLH family members altered the tolerance of mycorrhizal litchi to carbohydrate starvation (Shu et al., 2016). These results indicate that ERF, Myb, WRKY, bHLH and other transcription factors are involved in the response of mycorrhizal plants to different stresses, and play positive and negative regulatory roles. In this study, we found that the expression levels of several transcription factors in snapdragon would be significantly down-regulated under LT, WL, and LTWL stress, while the expression levels were up-regulated after AMF inoculation. Here, AMF inoculation and stress domestication caused some similar metabolic changes in plants. It is well known that plant domestication plays an important role in agricultural production, but the molecular mechanism of domestication is still not well understood. However, the molecular mechanism of domestication can be summarized as the accumulation of signaling proteins or transcription factors in an inactive state or the occurrence of epigenetic inheritance. During domestication, cells accumulate inactive proteins, which play an important role in signal amplification. When confronted with biological and abiotic stress, dormant signaling proteins are activated, and the initial signal is extended to activate stronger defense, immunity and stress resistance (Beckers et al., 2009).

AMF inoculation induced the expression of genes related to photosynthesis in snapdragon. Li et al. (2020) identified 24 genes related to photosynthesis and respiratory metabolism in mycorrhizal maize, and AMF inoculation could reduce the production and transfer of electrons in maize chloroplasts and mitochondria at LT. In addition, in this work, AMF inoculation significantly increased the transcription level of Rubisco protein under LT, and photosystem II related proteins under WL, possibly because AMF inoculation enabled the PSII center to capture more light energy would improve the photosynthetic efficiency and accelerate the recovery of PSII from photoinhibition more quickly (Endo et al., 2014). In addition, AMF inoculation induced the expression of genes related to active oxygen metabolism, and mycorrhizal blueberry induced the expression of some genes related to LT stress through activation of the antioxidant enzyme system, which resulted in resistance to LT (Liu et al., 2017; Tu et al., 2019), it has been reported that inoculation with *D. versiformis* can significantly enhance PtPOD and PtF-SOD in leaves as well as the expression levels of PtMn-SOD and PtCAT1 (Cao et al., 2022). Similar results were obtained in this study. Under LTWL conditions, the expression levels of two POD genes and one SOD gene were up-regulated. These findings indicated that AMF inoculation significantly enhanced the expression of antioxidase genes in snapdragon resulting in improved tolerances of snapdragon to LT, WL, and LTWL stress.

## 5 Conclusion

LT, WL, and LTWL stress inhibited the growth of the root system of snapdragon and caused oxidative stress by increasing the levels of reactive oxygen species and lipid peroxidation. However, inoculation with AMF reversed these phenomena and improved the resistance of snapdragon seedlings to LT, WL, or LTWL to some extent. Under LTWL condition, AMF inoculation acted by influencing osmotic regulatory substances (soluble sugars, soluble proteins, and proline), the activity of antioxidant enzymes (SOD, POD, CAT, APX, DHAR, and MDHAR), and contents of antioxidant substances (AsA, DHA, GSH, and GSSH). AMF also increased the expression of *bHLH112*, *WRKY72*, *MYB86*, *WRKY53*, *WRKY6*, *WRKY26* and *ARF5* and *ARF16* under LT, WL, and LTWL conditions, as well as genes related to photosynthesis, sugar transporters, all of which contributed synergistically to alleviating damage caused by LT, WL, and LTWL-induced oxidative stress. Thus, we inferred that inoculation with AMF enhances LTWL tolerance in snapdragon by regulating substance synthesis, transcriptional activation, membrane stability, and ROS clearance.

## Data availability statement

The original contributions presented in the study are included in the article/**Supplementary Material**. Further inquiries can be directed to the corresponding author.

## Author contributions

WL: Data curation, Formal analysis, Writing – original draft, Writing – review & editing. HW: Methodology, Writing – review & editing. JH: Resources, Writing – review & editing. CZ: Writing – review & editing. SG: Conceptualization, Writing – review & editing.
